# Endogenous expression and localization of HIS-72::mTurquoise2 in *C. elegans*

**DOI:** 10.17912/micropub.biology.000471

**Published:** 2021-09-29

**Authors:** Dillon E. Sloan, Joshua N. Bembenek

**Affiliations:** 1 University of North Carolina, Chapel Hill; 2 University of Michigan

## Abstract

To generate a non-red/green fluorescent fusion histone protein in *C. elegans*, we have generated a C-terminal mTurquoise2-tagged HIS-72 at the endogenous locus using CRISPR. We found that HIS-72::mTurquoise2 localizes in a similar pattern to the previously published HIS-72::GFP strain.

**Figure 1. Endogenous HIS-72::linker::mTurquoise2:**
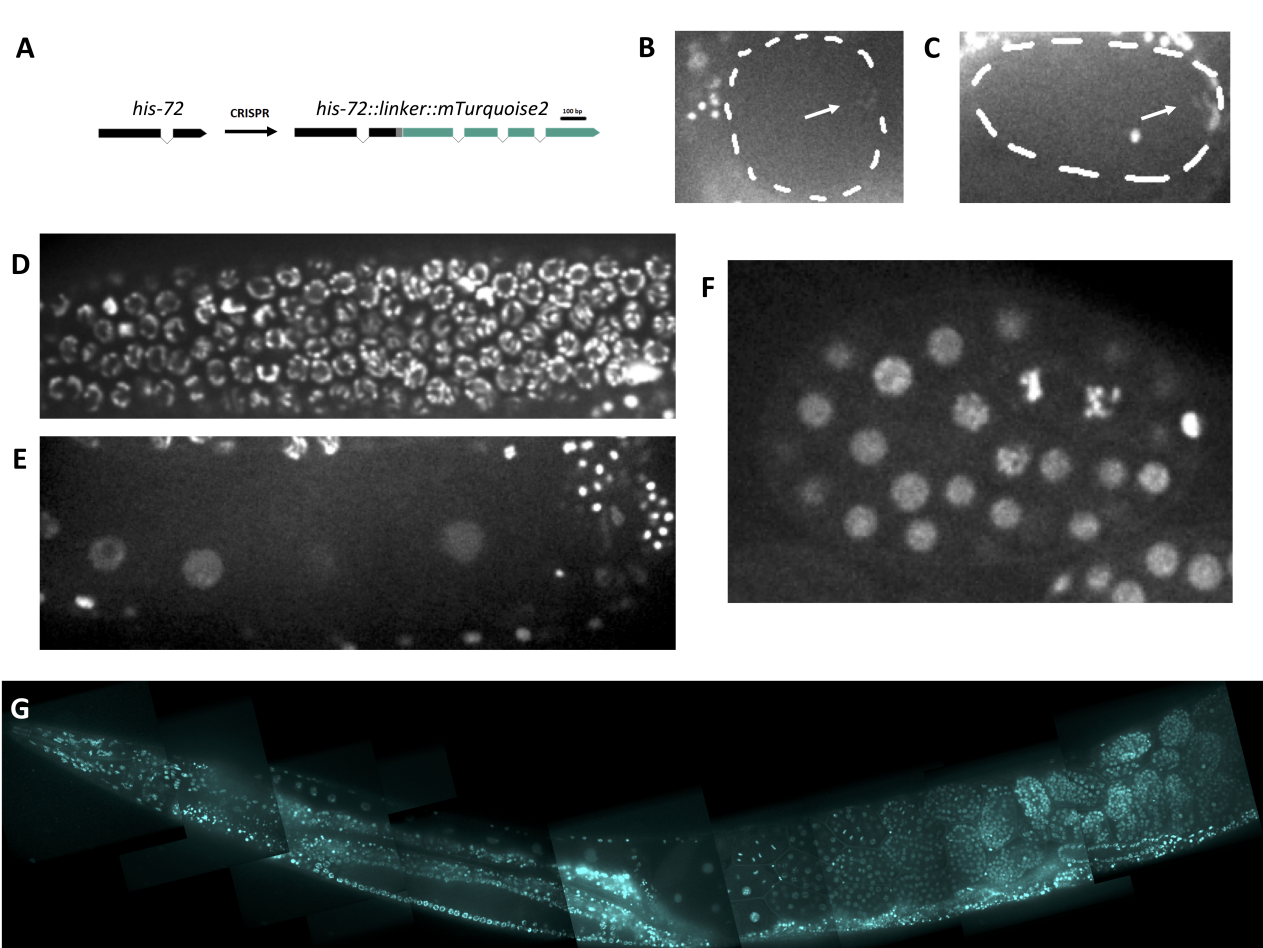
(A) Schematic of *his-72* edit (B-F) Images of HIS-72::linker::mTurquoise2 localization: in a prometaphase I embryo (B, arrow indicates meiotic chromosomes), in an anaphase I embryo (C, arrow indicates meiotic chromosomes), at the distal gonad arm (D), in the proximal gonad arm (E), and in a 50-100 cell embryo (F). (G) HIS:-72::linker::mTurquoise2 in an adult worm.

## Description

In fixed samples, chromatin is most often visualized by using a fluorescent DNA-binding dye like DAPI, which emits blue light (Kapuscinski 1995). In live fluorescence microscopy, however, a fluorescently tagged histone is often used instead. Histones form nucleosomes to package chromatin, and are involved in the organization of the genome (Chen *et al.* 2021). Most frequently, histones are fused to red or green fluorescent proteins, though the use of a blue or cyan fluorescent protein instead would allow for three (or more) channel imaging (Kanda *et al.* 1998, Das *et al.* 2003, Ooi *et al.* 2006).

We sought to generate an endogenously tagged histone line with a blue or cyan fluorescent protein. mTurquoise2 is a relatively recently engineered cyan fluorescent protein with a high quantum yield (Goedhart *et al*. 2012). Thus, we constructed a HIS-72::mTurquoise2 fluorescent fusion protein using a design similar to that of another group who also tagged HIS-72 (Figure 1A, Dickinson *et al.* 2013; see Design section). We report similar localization of HIS-72::mTurquoise2 to previously generated HIS-72 fluorescent fusion strains in most nuclei of the animal (Figure 1G). HIS-72::mTurquoise2 shows chromatin labeling in the germline and all stages of the cell cycle in the early embryo (Figure 1B-F). We observed that HIS-72::mTurquoise2 signal is significantly reduced on chromatin during the meiosis I and II divisions as compared with other stages (Figure 1B,C), which is not observed in other exogenously expressed fluorescently tagged histone lines (Bai and Bembenek 2017, Bembenek *et al.* 2007). This may reflect the role of different histone variants in meiotic chromosome function.

In sum, we have constructed a HIS-72::mTurquoise2 fluorescent fusion strain for use to the *C. elegans* community.

## Methods

*CRISPR/Cas9 Gene editing*

We followed the CRISPR/Cas9 protocol generated by the Seydoux lab for C-terminal mTurquoise2 tagging of the *C. elegans* his-72 gene (Paix *et al.* 2015). The repair template was amplified from the pDD377 plasmid, a gift from Bob Goldstein (Addgene plasmid #91823). Tagging of his-72 was done in the wild type N2 background. The transgenic strain was crossed to the N2 strain for 5 generations to remove potential off target mutations. All guide RNAs and oligos were obtained commercially.

The primer sequences for amplifying the repair template are listed below:

his-72::mTurquoise2_Forward (*DS_030*):

TGCAACTCGCCAGACGCATCAGAGGAGAACGTGCTGGAGCATCGGGAGCCTCAGGAGCATCGATGGTAAGTAAGGGCG

his-72::mTurquoise2_Reverse (*DS_031*):

ATTAAAAGTGCTTCGAGAATTGGTGATGGAGCTTACTTGTAGAGCTCGTCCATTCC

The flexible linker sequence: GGAGCATCGGGAGCCTCAGGAGCATCG (AA seq: GASGASGAS)

The target sequence (minus PAM) used in the crRNA (*Termed DS_g004; see Dickinson *et al.*, 2013*): GAGCTTAAGCACGTTCTCCG

NOTE: This design only tags HIS-72 isoform a (Y49E10.6a, see WormBase).

*Microscopy*

Gravid adult worms were mounted on agar pads according to standard methods. Imaging was performed on a spinning disk confocal system consisting of a Nikon Eclipse inverted microscope with a 60×1.40 NA objective, a CSU-22 spinning disk system, and a Photometrics EM-CCD camera from Visitech International. Images were acquired by Metamorph (Molecular Devices) and analyzed by ImageJ/Fiji Bio-Formats plugins (National Institutes of Health) (Schindelin *et al.* 2012). The cyan/blue channel included a 450/50 emission filter with maximum blocking at 405 nm, and maximum intensity from 425-475 nm.

## Reagents

JAB191: *his-72*(*erb77*[his-72::linker::mTurquoise2])

This strain will be made available to the CGC.
